# Liver Organoids: From Disease Modelling to Regenerative Medicine

**DOI:** 10.1111/cpr.70268

**Published:** 2026-08-14

**Authors:** Tiepeng Wang, Xiaotian Qu, Jinhong Si, Junkang Meng, Ting Zhang, Shuliang Niu

**Affiliations:** ^1^ Xinjiang Second Medical College Karamay Xinjiang China; ^2^ Carver College of Medicine, the University of Iowa Iowa City Iowa USA; ^3^ School of Medicine, Shihezi University Shihezi Xinjiang China

**Keywords:** disease modelling, drug screening, liver organoids, pluripotent stem cells, regenerative medicine

## Abstract

Liver organoids are three‐dimensional miniature liver models that recapitulate the complex architecture and key functions of the human liver in vitro, offering powerful platforms for both fundamental research and translational applications. This review systematically summarises current fabrication strategies, disease‐modelling utilities and regenerative potentials of liver organoids, alongside the major challenges and future directions. In recent years, the field has witnessed several breakthroughs. Through endothelial co‐culture approaches, vascularised and metabolically zonated liver organoids have been successfully generated, achieving endothelial coverage exceeding 85%. Prime editing enables precise correction of pathogenic mutations in patient‐derived organoids, with no off‐target effects detected at the genome‐wide level. In disease modelling, iPSC‐derived liver organoids faithfully recapitulate the pathological progression of metabolic dysfunction‐associated steatotic liver disease (MASLD) and verify the lipid‐lowering efficacy of semaglutide. Macrophage‐integrated organoid models support the full life cycles of HEV, SARS‐CoV‐2 and dengue virus, providing new tools for antiviral drug screening. Large‐scale patient‐derived tumour organoid biobanks successfully preserve the heterogeneity and clinical drug‐resistance signatures of liver cancers. In regenerative medicine, encapsulated hepatocyte organoids and the UTOpiA bioartificial liver system have effectively rescued acute liver failure in animal models, while gene‐edited autologous organoids offer potential curative strategies for genetic disorders such as Wilson disease. Nevertheless, insufficient hepatocyte functional maturity, difficulties in constructing vascular networks, and the lack of standardised culture protocols remain major obstacles to clinical translation. By bridging fundamental liver biology and clinical practice, liver organoid technology lays a solid foundation for precision hepatology and regenerative therapies. Continued interdisciplinary efforts are still required to overcome current limitations and facilitate its clinical adoption.

## Graphical abstract

Liver organoids provide a versatile platform for disease modelling and drug discovery, leveraging stem cells and engineering techniques. They bridge research and clinical applications, offering significant potential for advancing precision medicine and regenerative therapies for liver diseases.

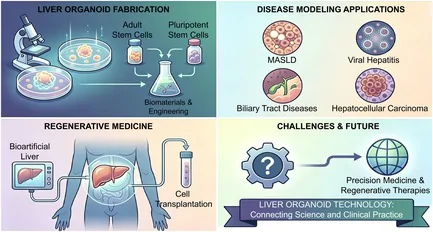

## 1 Introduction

The liver, as a vital organ in the human body, possesses a complex structure and diverse functions, including metabolic regulation, detoxification, protein synthesis and bile secretion. Liver diseases—including viral hepatitis, metabolic dysfunction‐associated fatty liver disease, cirrhosis and hepatocellular carcinoma—have become a major global health burden, causing millions of deaths annually [1]. Currently, liver transplantation remains the only curative treatment for end‐stage liver disease. However, its application is severely constrained by a critical shortage of donor organs [2]. Traditional liver research models rely on simplified two‐dimensional cell cultures and animal models. Although numerous targeted genes and signalling pathways have been reported, significant challenges persist in clinical translation and application. Therefore, developing novel liver disease models and innovative therapeutic strategies is of paramount importance.

Organoids are three‐dimensional (3D) miniature organs derived from stem cells or tissue‐specific progenitor cells. They spontaneously self‐organise in vitro, reproducing the cellular composition, spatial architecture and physiological functions of their donor organs [3, 4]. Since the first successful cultivation of intestinal organoids in 2009 [5], this technology has expanded to encompass multiple organ systems, including the liver. Given the liver's exceptional regenerative capacity compared to other tissues and organs, the development of liver organoids holds distinct advantages over other organoid types.

The field of liver organoids has evolved through several stages. In 2013, the Clevers team established the first long‐term culture of mouse liver organoids, providing a foundational proof‐of‐concept [6]. This was followed in 2015 by their derivation of genetically stable, bipotent hepatic progenitor cells from adult human liver, capable of differentiation into functional hepatocytes and cholangiocytes [7]. By 2018, methodologies expanded as multiple groups achieved long‐term 3D organoid culture from both mouse and human primary hepatocytes, significantly broadening the platform's applicability [8, 9]. In recent years, advances in single‐cell sequencing, gene editing and bioengineering technologies have collectively propelled progress in liver organoid research, giving rise to more sophisticated models [10, 11, 12]. Today, next‐generation organoids can integrate multiple cell types and begin to replicate the liver's complex metabolic compartmentalisation, providing a more ‘realistic’ research system for disease modelling and therapeutic development.

Liver organoid technology has the potential to support a range of applications, from basic research to clinical translation. In disease research, this technology can closely recreate hereditary liver diseases, infectious diseases like viral hepatitis and malignant tumours like hepatocellular carcinoma, providing a very accurate model for understanding the causes of diseases and testing a lot of drugs at once [13, 14]. In regenerative medicine, liver organoids have two important uses. They can be used to make transplantable liver cells. They can also be used in bioartificial liver (BAL) systems. This offers new hope for patients with end‐stage liver disease [15, 16]. This technology also helps us understand important biological processes like liver development, how tissues repair themselves and how the liver works with other organs.

This review aims to provide a thorough summary of the development of liver organoid technology, with a focus on its applications in disease modelling and regenerative medicine. We will look at the current challenges and plan for the future. This will be a useful resource for researchers and will help liver organoid technology advance in basic and translational research.

## 2 Construction Strategies and Technological Advances

There are various strategies for constructing liver organoids, which are primarily determined by cell sources, culture conditions and intended applications. Liver organoids can be classified based on cell origin as those derived from adult stem cells or pluripotent stem cells [17]. Furthermore, integrating biomaterials and engineering technologies has enhanced their complexity and functionality.

### 2.1 Adult Stem Cell‐Derived Liver Organoids

Adult liver stem cells, particularly LGR5^+^ cells found in bile ducts, have the ability to self‐renew and differentiate into two types of cells: hepatocytes and cholangiocytes [5]. In 2015, Huch et al. first reported the long‐term culture of genetically stable, bipotent stem cells from the adult human liver that could form organoids in vitro and differentiate into functional hepatocytes [6]. Similarly, Hu et al. developed a 3D organoid culture system in 2018 that can expand functional mouse and human hepatocytes long term [18].

The standard process for creating liver organoids using adult stem cells usually involves collecting stem/progenitor cells from liver tissue samples, placing them in a special liquid (like Matrigel) and growing them in a medium with specific growth factors (like EGF, R‐spondin 1, FGF10 and HGF) [19, 20]. In these conditions, cells organise themselves into 3D structures while keeping their ability to specialise. The advantages of this method include: (1) It keeps the genetic information and epigenetic background of the source tissue, making it a great tool for creating personalised disease models; (2) It can expand a lot, which supports long‐term culture; (3) It can incorporate different types of hepatocytes, like hepatocyte‐like and cholangiocyte‐like cells [21]. But there are also some clear limitations: (1) The cells are often not fully mature, and they look more like cells found in an embryo or during regeneration than fully mature liver cells. (2) Other types of cells, like endothelial cells and stellate cells, are usually not present. (3) The use of animal‐derived materials limits the potential for clinical applications.

### 2.2 Pluripotent Stem Cell‐Derived Liver Organoids

Human pluripotent stem cells (hPSCs), including embryonic stem cells (ESCs) and induced pluripotent stem cells (iPSCs), can make any type of cell [22]. This makes them an ideal source for constructing liver organoids. hPSC‐derived liver organoids are typically generated by recreating liver development. First, hPSCs are changed into definitive endoderm. Then, they are changed into hepatic progenitors. Finally, they are further differentiated and organised into organoids in 3D culture [23].

Recently, various complex hPSC‐derived liver organoid models have emerged. For instance, in 2022, Wesley et al. mapped human liver development at single‐cell resolution and used this information to guide the generation and functional maturation of hepatoblast organoids [24]. In 2024, Carolina et al. successfully generated liver organoids incorporating 3D bile duct structures by co‐culturing hPSC‐derived liver progenitors with hPSC‐derived blood vessels [25]. Takebe et al. developed a liver bud system by co‐culturing hepatic endoderm, mesenchymal stem cells and endothelial cells, generating organoids with vascular features [26]. The benefits of organoids derived from hPSCs are: (1) they can replicate developmental stages; (2) they can incorporate various cell types to achieve greater complexity; and (3) they provide an unlimited supply without relying on donor tissues. However, there are still obstacles to overcome, including: (1) ensuring efficient differentiation and maintaining quality control; (2) considerable variability between batches; and (3) the requirement for enhanced functional maturity.

### 2.3 Biomaterials and Engineering Strategies

To overcome the limitations of regular organoid culture, different biomaterial and engineering strategies have been developed to improve the functionality and reproducibility of liver organoids (Figure 1).

**FIGURE 1:**
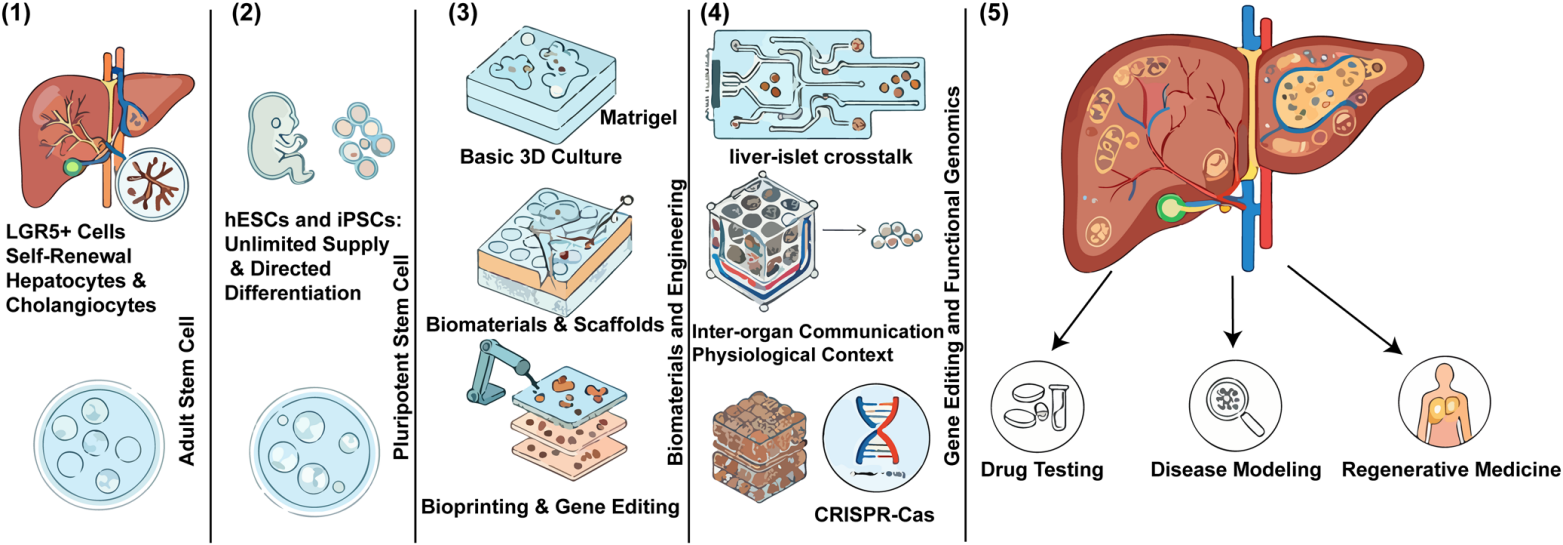
Biomaterials and engineering strategies for the establishment of liver organoids. (1) Adult stem cell source: Resident LGR5^+^ hepatic stem cells in native liver possess self‐renewal capacity and can differentiate into functional hepatocytes and cholangiocytes for organoid construction. (2) Pluripotent stem cell source: Human embryonic stem cells (hESCs) and induced pluripotent stem cells (iPSCs) provide unlimited cell resources, which can undergo directed hepatobiliary differentiation to generate standardised liver organoids. (3) Biomaterials and bioengineering toolkit: Multiple culture systems support organoid maturation, including basic Matrigel‐based 3D culture, composite biomaterial scaffolds, 3D bioprinting and gene editing modification. These technologies reconstruct the physiological microenvironment of liver tissue in vitro. (4) Inter‐organ co‐culture and functional genomics: Advanced platforms integrate liver organoids with other parenchymal organ units (e.g., pancreatic islets) to simulate inter‐organ crosstalk; combined with CRISPR‐Cas gene editing, they enable high‐throughput functional genomic screening. (5) Translational clinical applications: Mature liver organoid systems serve three major research directions: Preclinical drug toxicity testing, humanised disease modelling for pathological mechanism exploration and regenerative medicine for liver tissue repair and transplantation.

Synthetic hydrogels are becoming more popular because they are made from non‐animal sources, which are more readily accessible. For example, Ye et al. developed a hydrogel based on polyisocyanopeptides (PICs) and laminin‐111 that supported the long‐term culture and differentiation of human liver organoids (HLOs) [19]. Sorrentino et al. used chemically defined hydrogels to make liver organoids in mice and humans. They found that the organoids' growth was really sensitive to the stiffness of the matrix [27]. Wang et al. reported a synthetic click hydrogel that could be tuned to control the hepatic lineage specification of HLOs [28].

Vascularisation strategies are key to improving the functional maturity and transplant survival of liver organoids. Zhu et al. developed vascularised liver microtissues by culturing liver organoids along with vascular organoids. This approach promoted the structural and secretory maturation of hepatocytes via paracrine signalling from the vasculature [29]. Fan et al. presented a non‐parenchymal cell grafting (NCG) strategy to form pre‐vascularised human hepatobiliary organoids on a large scale without affecting liver epithelial specification [30]. Zhang et al. generated liver organoids exhibiting zonation features through the co‐culture of human embryonic stem cell (hESC)‐derived hepatocytes and liver sinusoidal endothelial cells (LSECs) [31].

Without co‐cultured endothelial cells or bioengineered vascular channels, standard liver organoids fail to generate perfusable vascular networks, with fewer than 10 CD31‐positive luminal structures per square millimetre of tissue, and the maximum culture size is limited to 200–500 μm due to central cell necrosis. After introducing vascularisation engineering strategies, endothelial coverage can reach 85% ± 5%, and the barrier permeability of engineered microvessels is close to native liver sinusoidal endothelium [32].

Microfluidics and organ‐on‐a‐chip technologies provide a more physiologically relevant environment for liver organoids. Tao et al. developed a microengineered multi‐organoid system to recapitulate the human liver‐islet axis under normal and type 2 diabetic conditions [33]. Li et al. constructed a microfluidic human liver‐islet crosstalk platform for modelling MASLD [34]. These systems enable the co‐culture of multiple organoid types and the study of inter‐organ communication, offering new platforms for systemic disease modelling.

Bioprinting technologies enable the high‐throughput production and precise assembly of liver organoids. Bernal et al. reported the volumetric bioprinting of liver organoids and optically tuned hydrogels to build liver‐like metabolic biofactories [35]. Luo et al. developed a method for creating rapid self‐assembly mini‐livers (RSALs) within 12 h, which were functionally vascularised upon transplantation and protected mice from hepatectomy‐induced liver failure [36].

### 2.4 Gene Editing and Functional Genomics

CRISPR‐Cas9 technology has made it much easier to change the genes of liver organoids and create models of diseases. Artegiani et al. developed a new method called CRISPR‐HOT (CRISPR‐Cas9‐mediated homology‐independent organoid transgenesis) that allows for efficient gene insertion in human organoids without the need for extensive cloning [11]. Schene et al. used a special kind of gene editing called ‘prime editing’ to fix the problem in patient‐derived disease models. This showed that prime editing can be used as a treatment for diseases [37]. Geurts et al. showed how base editors can be used to make complex tumour models in human adult stem cell‐derived organoids in just one step [38].

Single‐cell multi‐omics analysis gives researchers powerful tools for understanding the variety of cells and molecules in liver organoids. Wesely et al. created a map of how the human liver develops. They did this using a process that examines the genetic code of a cell at the single‐cell level. This map helps scientists create miniature versions of the liver called ‘organoids’ [24]. Hendriks et al. performed a type of analysis that looked at the early stages of organoid growth from foetal and adult hepatocytes. This research provides insights that can help improve maturation and expansion [39].

In summary, while the advancement of liver organoids has enabled researchers to create increasingly complex and functional liver models, providing powerful tools for disease research, drug testing and regenerative medicine studies, limitations persist. These include incomplete cellular maturity and reliance on animal‐derived materials in adult stem cell‐derived organoids; inefficient differentiation, batch variability and inadequate functional maturation in pluripotent stem cell‐derived counterparts; challenges in biomaterial biocompatibility and biological mimicry; critical bottlenecks in vascularisation (e.g., lack of functional sinusoidal networks and hepatic zonation); scalability and technical complexity issues in engineering technologies like bioprinting and microfluidics; suboptimal efficiency and precision in organoid gene editing; and translational hurdles such as regulatory barriers, immunogenicity risks and the absence of standardised protocols and quality control metrics.

## 3 Applications in Disease Modelling

Liver organoids can copy the normal and abnormal functions of the human liver. This makes them a useful tool for studying diseases, testing drugs and creating personalised treatment plans. Figure 2 summarises the full technological workflow and translational applications of liver organoid platforms, covering the source cell acquisition, biomaterial fabrication, multi‐organ co‐culture engineering, gene editing toolkit and three core translational branches: drug testing, disease modelling and regenerative medicine. The schematic is divided into five sequential modules as illustrated below:

**FIGURE 2:**
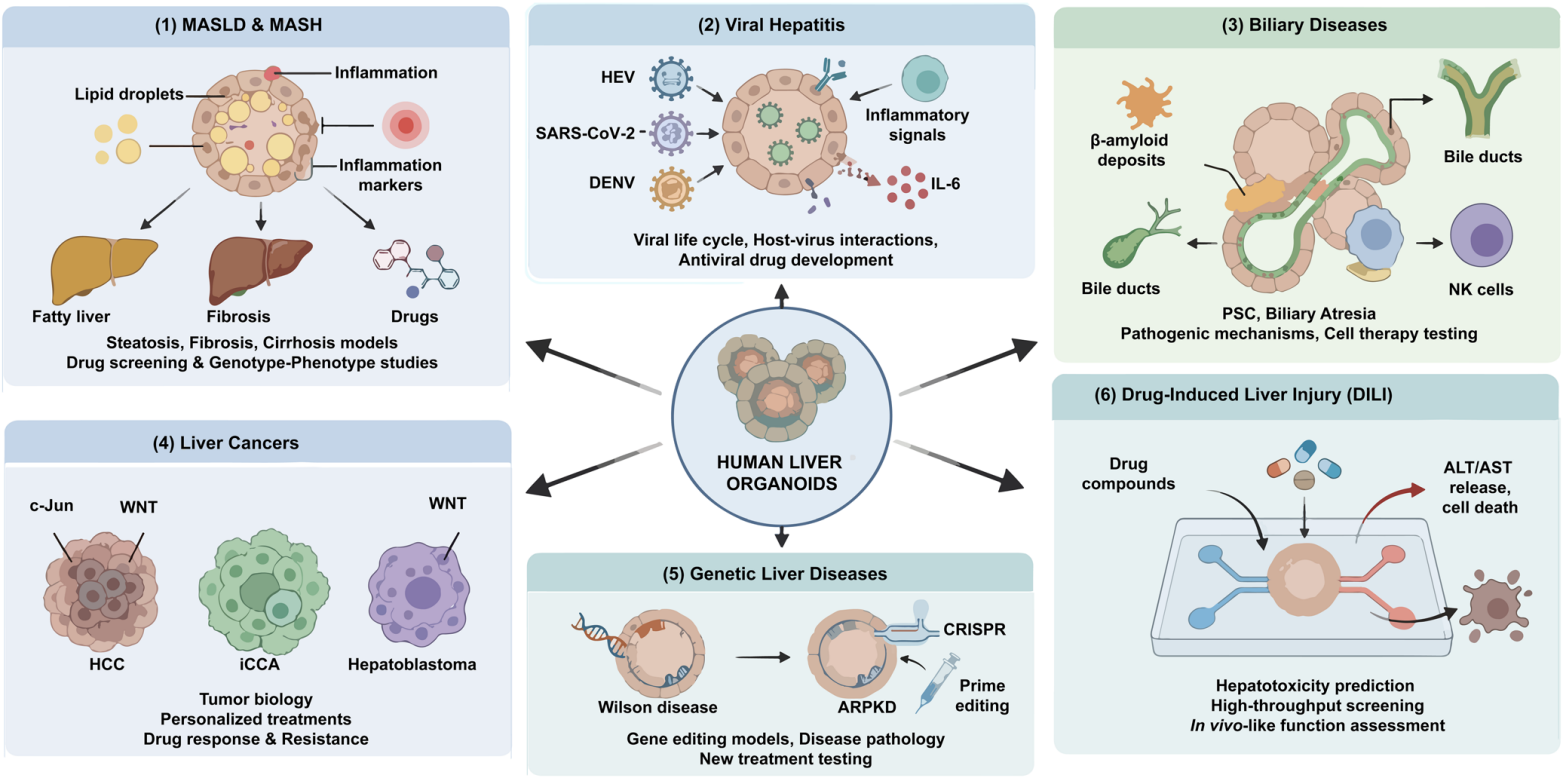
Applications of liver organoids in disease modelling. (1) MASLD and MASH modelling: Organoids recapitulate steatosis, fibrosis and inflammation, supporting drug screening and genotype–phenotype research. (2) Viral hepatitis research: Supports complete viral replication of HEV, SARS‐CoV‐2 and DENV to study host‐virus interaction and antiviral drug development. (3) Biliary disease modelling: Reconstruct bile duct lesions of PSC and biliary atresia to explore pathogenesis and test cell therapy. (4) Liver cancer modelling: Tumour organoids retain HCC, iCCA and hepatoblastoma oncogenic signals for personalised therapy and drug resistance study. (5) Genetic liver disease modelling: Combined with CRISPR/prime editing to build Wilson disease and ARPKD models for pathology and therapeutic testing. (6) Drug‐induced liver injury evaluation: Microfluidic organoid platform detects ALT/AST release and hepatocyte death for high‐throughput hepatotoxicity screening.

### 3.1 MASLD and Metabolic Dysfunction‐Associated Steatohepatitis (MASH)

The most common chronic liver disease in the world is MASLD. It is characterised by the buildup of excess fat in the liver cells. This can lead to MASH, fibrosis and cirrhosis. Liver organoids are useful for studying the causes and treatment of fatty liver disease [40, 41].

Hendriks et al. used human foetal hepatocyte organoids to model early MASLD (steatosis) with three triggers: free fatty acid (FFA) loading, PNPLA3 I148M genetic variability and APOB/MTTP monogenic lipid disorders. Drug screening identified steatosis‐resolving compounds via convergent de novo lipogenesis repression; they further developed FatTracer, a CRISPR platform for steatosis modulator screening [42]. Kimura et al. created a group of human organoids to study how metabolic status affects genotype–phenotype associations. They found that a specific genetic variation increases disease severity in people with diabetes but protects against fibrosis in people without diabetes. Gene expression, metabolite and pharmacological studies showed significant mitochondrial dysfunction from this variation, which metformin did not reverse [43]. Osonoi and Takebe reviewed organoid‐guided precision hepatology for metabolic liver disease, emphasising the integration of synthetic tissue models of key metabolic organs. By combining functional genomics and population‐scale profiling, patient‐derived tissues can provide insights into personalised states [44]. Liu et al. investigated the therapeutic implications of ACMSD inhibition in MASLD/MASH. Inhibiting ACMSD promoted de novo NAD^+^ synthesis and reduced DNA damage. Therapeutic inhibition in mice increased liver NAD^+^ and reversed MASLD/MASH, mitigating fibrosis, inflammation and DNA damage, as also observed in HLO models of steatohepatitis [45]. Besides the aforementioned disease models, liver organoids serve as a robust preclinical drug evaluation platform for non‐alcoholic fatty liver disease (NAFLD). A study used hiPSC‐derived liver organoids (hLOs) induced by FFAs to mimic NAFLD hallmarks (steatosis, inflammation and fibrogenesis). Treatment with 50 nM semaglutide (a GLP‐1 receptor agonist) significantly reduced FFA‐induced lipid deposition, lowering triglyceride and cholesterol levels by 8‐fold and 1.8‐fold, respectively [46].

Taken together, these studies underscore the profound potential of liver organoids in elucidating the pathogenesis of 60, facilitating drug screening and discerning genotype–phenotype associations that are instrumental for patient stratification. However, the absence of native hepatic complexity imposes limitations on the recapitulation of advanced disease stages. Consequently, the integration of organoids with complementary technologies will be pivotal to the advancement of precision hepatology for metabolic liver diseases.

### 3.2 Viral Hepatitis

Viral hepatitis is a major cause of liver‐related deaths around the world. Liver organoids are an important tool for studying how viruses work, how they interact with their hosts and how to develop antiviral drugs.

For hepatitis E virus (HEV), Liu et al. studied special cell cultures called ‘iPSC‐induced human liver, intestinal and brain organoids’ to learn how they could be used to study HEV infection. All organoids supported the complete HEV life cycle. Liver organoids showed signs of infection in certain cells, along with higher than normal levels of a protein called IL‐6 and impaired liver function [47]. For SARS‐CoV‐2, Yang et al. created a special system using special cells called hPSCs to study how the virus spreads. They found that human pancreatic beta cells and liver organoids were very susceptible to SARS‐CoV‐2 infection, which caused the cells to produce a lot of chemokines, as seen in samples from people with severe cases of SARS‐CoV‐2 [48]. Liu et al. created a special type of organoid called MaugOs. These organoids have macrophages inside them. When infected with HEV, SARS‐CoV‐2, or monkeypox virus, MaugOs recreated the infection and the resulting inflammation, creating a model for how immune cells can cause disease [49]. Li et al. used special liver cells to study the effects of the dengue virus. They found that the cells were very sensitive to the virus, leading to significant cell death. Using a special technique that looks at genes in single cells, they saw that the liver cells were the main targets of the virus, and the virus caused a lot of damage to the cells' mitochondria. Drug screening found oxyresveratrol and omaveloxolone, which are strong candidates for fighting against DENV. These drugs activated the NRF2 pathway [50].

All of these studies show that organoid models are better at showing how viruses affect cells, how the body's immune system responds to them and how the pathogens cause tissue damage. This is something that is often not considered when using traditional cell lines. These advanced platforms help us understand viral hepatitis and cross‐species viral infections better. They also help us find new ways to treat viral infections.

### 3.3 Biliary Diseases

Biliary diseases, like primary sclerosing cholangitis (PSC) and biliary atresia (BA), are a big reason why people have liver failure. Liver organoids are special because they can help scientists study how the liver works and develop new treatments.

Researchers report the creation of mouse liver and bile duct organoids using liver cell building blocks and bile duct cells. These organoids show how liver cells (forming networks to produce bile and maintain long‐term metabolic functions) and bile duct cells (forming tubes to transport bile) work together [51]. Sampaziotis et al. showed that liver cells can repair bile ducts after transplantation in human livers using a special machine to circulate the liver outside the body at normal body temperature. This provides a way to test the use of cell therapy for diseases of the bile ducts [16]. Babu et al. used organoids from BA patients and a mouse model. They discovered that a substance called beta‐amyloid builds up around bile ducts. This is a new way to understand the disease. Beta‐amyloid exposure causes abnormal organoid structure, revealing a new pathogenic mechanism in BA [52]. Zecher et al. found that a specific combination of genes, called HLA‐DPA1*02:01∼B1*01:01, increases the risk of PSC by causing NKp44^+^ NK cells to become active. Human cholangiocyte organoids with this genetic variation increased the release of chemicals from NK cells, suggesting that this interaction contributes to the development of PSC [53]. Defamie et al. discovered that a group of proteins called the TIMP/ADAM/NOTCH/DLK1 axis plays a key role in maintaining liver progenitor cells (LPCs). TIMP‐deficient biliary organoids showed increased differentiation of cholangiocytes. Treatment with soluble DLK1 triggered the expression of a protein called Sox9 and helped to specify the cells that form the bile ducts in mouse liver cells and liver organoids from patients [54].

These breakthroughs show that patient‐derived and genetically engineered liver organoids are powerful tools for understanding the complex causes of biliary diseases and confirming new ways to treat them. While these organoid models have revealed important details about the causes of biliary diseases, a big challenge remains: can the simplified in vitro microenvironment of organoids fully recreate the complex interactions between bile ducts, liver stromal cells and the immune system observed in human patients?

### 3.4 Liver Cancers

Liver cancers, including hepatocellular carcinoma (HCC), intrahepatic cholangiocarcinoma (iCCA) and hepatoblastoma, are very different from each other and are hard to treat. Patient‐derived tumour organoids (PDTOs) are a useful tool for studying tumour biology and developing personalised treatments.

Yang et al. created a collection of 399 tumour organoids (small samples of tumours) from 144 patients with primary liver cancer (PLC). These organoids showed the same characteristics as the patients' original tumours and had the same genetic changes. They used a method called ‘integrative analysis’ to study how different people's cells respond to drugs and how they change over time. This showed that a protein called c‐Jun helps some cells resist a drug called lenvatinib [55]. Lee et al. confirmed two iCCA subgroups that had recently been suggested (small‐duct and large‐duct) using organoid models [56]. Kluiver et al. did a complete study of hepatoblastoma and tumour‐derived organoids. They found two different types of tumour epithelial signatures: hepatic ‘foetal’ and WNT‐high ‘embryonal’, which show different WNT signalling. Embryonal and foetal tumour organoids were sensitive to FGFR and EGFR inhibitors, respectively, indicating a dependency on EGF/FGF signalling [57]. Li used a special type of organoid that came from both the original tumour and the liver metastasis in the same patient with colorectal cancer. Even though the genes expressed were similar, organoids from metastatic lesions showed more aggressive characteristics, tumour formation and the ability to spread. Studies of gene activity revealed specific genes, like SOX2, that are linked to the development of CRC [58].

Liver cancer organoids can recreate tumour characteristics, but we still need to know how to add key parts of the tumour environment, like stromal cells, immune cells and vascular structures, to these models. The next step would be to add these elements to organoid systems. This would make the models better at predicting how drugs will act in a living body and how liver cancer will spread.

### 3.5 Genetic Liver Diseases

Some genetic liver diseases, like Wilson disease, Alpha‐1 antitrypsin deficiency and Alagille syndrome, can affect the liver from birth. Liver organoids are like tiny labs that allow scientists to study and test new treatments for these diseases. Schene and colleagues used a special kind of gene editing called “prime editing” to fix a disease in patient‐derived models. This included liver organoids from a patient with Wilson disease (ATP7B). This showed that this gene editing method can be used to treat diseases with minimal side effects [37]. Guan et al. created liver organoids that carry a common mutation associated with a rare kidney disease called autosomal recessive polycystic kidney disease (ARPKD). These organoids developed key features of ARPKD liver pathology (abnormal bile ducts and fibrosis) within 21 days. The mutation increased the activity of the TGFβ pathway in the cells of the bile ducts, which caused them to produce more collagen [59]. Cristinziano et al. expressed four different iCCA fibroblast growth factor receptor 2 (FGFR2) fusion proteins in Tp53^−/−^ mouse liver organoids. This successfully modelled the pathogenesis of iCCA driven by FGFR2. Tumour organoids were dependent on FF signalling via Ras–Erk, regardless of the type of FF [60]. Rüland et al. used special cells, called ‘CRISPR‐engineered human hepatocyte organoids’, to recreate different genetic backgrounds of a rare liver cancer called fibrolamellar carcinoma (FLC). When BAP1 and PRKAR2A were both lost, liver cells changed into cells that can form ducts or progenitor cells. This showed us more about how FLC tumours are formed [61].

Collectively, these studies highlight the unique value of liver organoids. However, it is also clear that we have not yet fully explored the potential of liver organoid models in this field. This means there is still room to improve the culture systems, expand the diseases that we study, and apply the results of our studies to patients.

### 3.6 Liver Organoids and Neurodegenerative Diseases

In recent years, increasing evidence has shown that there is a close physiological and pathological relationship between the liver and the brain [62, 63, 64]. Liver organoids, as powerful tools for simulating liver function, not only aid in liver disease research but can also be used to explore the connection between the liver and neurodegenerative diseases. For instance, the metabolic functions of the liver are closely linked to the health of the nervous system [65, 66, 67]. When the liver produces excessive toxic metabolites, such as ammonia, it can lead to brain dysfunction, which is notably seen in hepatic encephalopathy (HE). Studies suggest that liver organoids can be used to model these metabolic abnormalities and further investigate the relationship between the liver and neurodegenerative diseases [68, 69, 70]. Additionally, the liver is the source of many important biomarkers, which may play a key role in the early diagnosis of neurodegenerative diseases. For example, liver‐derived neurotoxic substances may cross the blood–brain barrier and trigger or exacerbate the pathological processes of neurodegenerative diseases [71, 72]. Research on liver organoids can provide new disease models to assess the impact of these neurotoxic substances and offer a foundation for the development of neuroprotective therapies.

Currently, combining liver organoids with models of neurodegenerative diseases, especially in HE, helps scientists better understand the brain function changes induced by liver dysfunction. These may offer new therapeutic strategies for patients with liver failure, improving cognitive and neurological impairments caused by liver dysfunction.

The above discussion focuses on endogenous liver dysfunction‐driven systemic and neurological lesions represented by HE and related neurodegenerative complications. Correspondingly, exogenous chemical stimuli represented by pharmaceutical compounds can also directly disrupt hepatocellular homeostasis and trigger liver damage. Endogenous liver metabolic disorders and exogenous drug‐induced liver injury (DILI) constitute two core parallel research directions of liver organoid modelling, both relying on HLO platforms to dissect pathological mechanisms and evaluate intervention safety and efficacy [73]. The following section will elaborate on the application of liver organoids in evaluating DILI and other infectious liver disease models.

### 3.7 DILI and Others

DILI is a common reason why drugs are not successful. It is also a common reason why drugs are removed from the market after they are released. This shows that we need better models to predict these problems.

Zhang et al. evaluated HLOs to see if they could predict the risk of DILI quickly and efficiently. Dispersed HLOs from three iPSC lines had similar predictive capacity in a high‐throughput format [74]. Optimised microfluidic liver organoids exhibit elevated CYP450 expression and albumin secretion, and their EC_50_ values for classic hepatotoxic agents deviate less than 20% from primary hepatocytes, supporting reliable high‐throughput hepatotoxicity prediction [75]. This demonstrates that they enhance in vivo‐like functions for DILI assessment. Researchers also confirmed that hepatocyte organoids (HepOrgs) support *Plasmodium falciparum* (Pf) invasion, differentiation and maturation. Single‐cell mRNA sequencing identified 80 upregulated Pf transcripts in infected HepOrgs on Day 5 [76].

In summary, liver organoids are a useful tool for studying a variety of liver diseases. They can provide new information about how diseases work and help develop new treatments. As technology keeps getting better, liver organoids are going to be very important for personalised medicine and drug discovery.

## 4 Potential in Regenerative Medicine and Cell Therapy

Liver organoids have great potential in regenerative medicine and cell therapy. With proper protocols, they can be used as a cell source, parts of BALs, or tissue engineering constructs, offering new hope for treating end‐stage liver disease (Figure 3).

**FIGURE 3:**
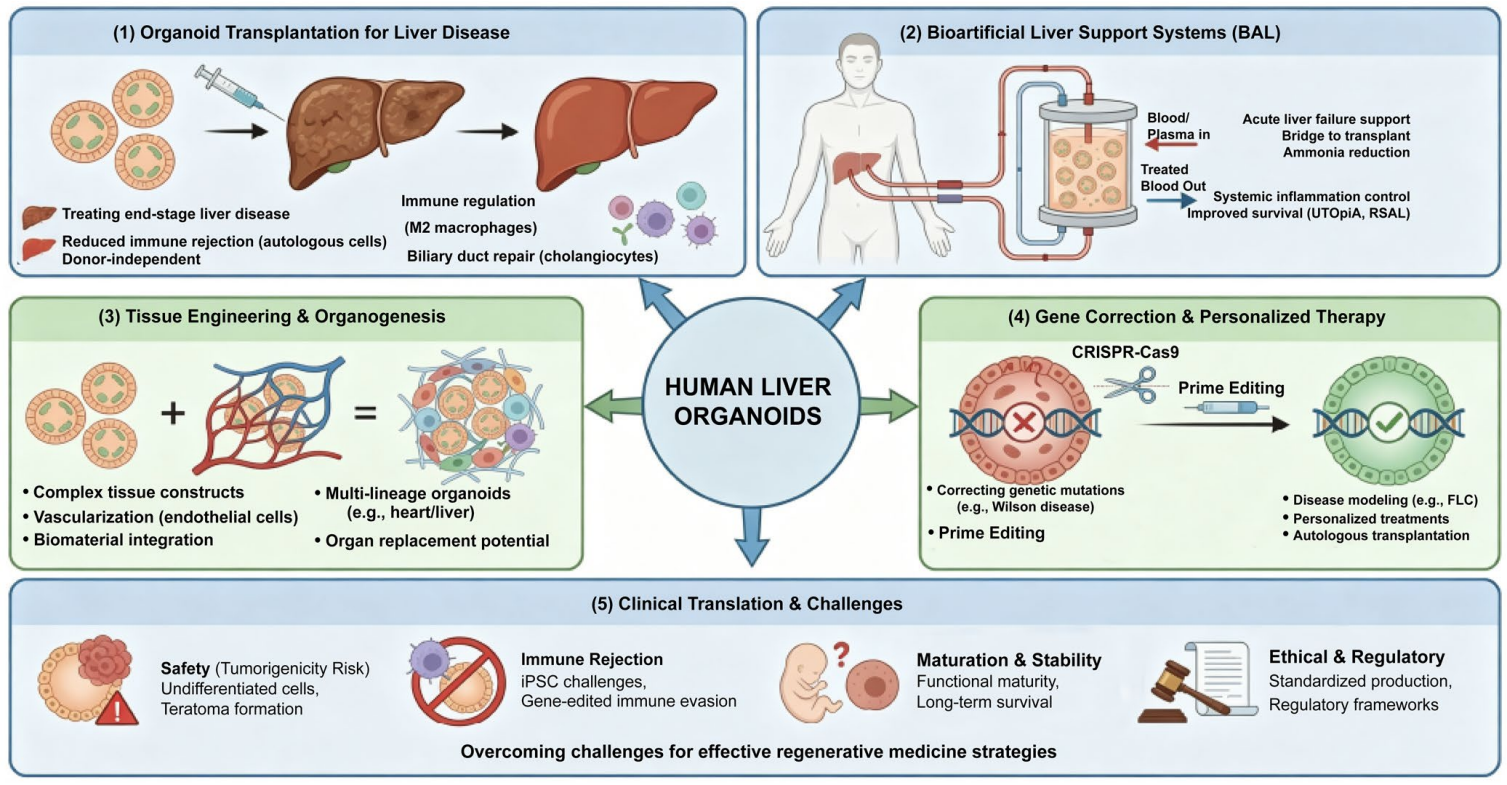
Liver organoids for regenerative medicine and cell therapy applications. (1) Organoid transplantation for liver disease: Autologous liver organoid injection treats end‐stage liver disease, avoids donor shortage and allogeneic rejection; therapeutic effects rely on M2 macrophage immune regulation and cholangiocyte‐mediated biliary duct repair. (2) Bioartificial liver (BAL) support systems: Extracorporeal devices loaded with liver organoids process patient blood/plasma to temporarily compensate liver function for acute liver failure, serving as a transplant bridge via ammonia removal, systemic inflammation suppression and improved survival supported by UTOpiA and RSAL data. (3) Tissue engineering and organogenesis: Combination of liver organoids with biomaterial scaffolds and endothelial cells yields vascularised, multi‐lineage complex hepatic constructs, holding potential for whole liver replacement and multi‐organ assembloid fabrication. (4) Gene correction and personalised therapy: CRISPR‐Cas9 and prime editing correct pathogenic mutations (e.g., Wilson disease) in patient‐derived liver organoids; edited autologous organoids support monogenic liver disease modelling, customised therapeutic design and mutation‐corrected cell transplantation. (5) Clinical translation and core challenges: Key barriers limiting clinical application include tumorigenic risks from undifferentiated cells, iPSC‐associated immune rejection, insufficient functional maturation and poor long‐term stability of organoids, plus unresolved ethical issues and absent unified regulatory standards for standardised organoid production.

### 4.1 Organoid Transplantation for Liver Disease

Organoid transplantation is a promising strategy for treating end‐stage liver disease. Compared to traditional liver transplants, it has some key benefits: it can potentially avoid immune rejection (using the patient's own cells), it does not rely as much on donor organs, and it can be delivered in a way that is less invasive.

Studies on liver organoids in animals have shown that they can help treat some diseases. Hu et al. showed that human liver cells transplanted into mice grew extensively after the cells were put into the mice, which is similar to how human liver cells behave when they are damaged [18]. Yuan et al. showed that alginate‐encapsulated proliferating human hepatocyte organoids (eLO) are safe and effective in treating liver failure in mice. Treatment with eLO increased survival in mice with liver failure after a liver transplant and improved problems like high ammonia and low blood sugar by replacing liver function [77]. Tadokoro et al. showed that transplanting hiPSC‐liver organoids (hiPSC‐LOs) reduced fibrosis by regulating the immune system. They found that the foetal liver strongly expressed factors that encourage the presence of macrophages and reduce scarring. Transplanting hiPSC‐LOs improved liver fibrosis caused by chemicals by regulating the immune system, especially on CD163^+^ phagocytic M2‐macrophage polarisation [78]. For biliary diseases, Sampaziotis et al. showed that cholangiocyte organoids can repair human intrahepatic ducts after transplantation in an ex vivo perfusion model, highlighting their potential for cell therapy of cholangiopathies [16]. Studies have shown that liver organoids derived from needle biopsies (b‐Orgs) can retain disease stage‐specific features and recapitulate the heterogeneity of liver epithelial cells. This model provides a new platform for investigating alcohol‐associated hepatitis [79].

In general, liver organoid transplantation has the potential to transform the treatment of advanced liver and biliary diseases. It could overcome the challenges of not having enough donors and the body's rejection of the transplanted liver. However, there are still important problems that need to be solved before this treatment can be used in clinics. These include improving the maturation of the organoids and making sure they function well in the body. It is also important to create standard ways to produce large amounts of organoids and to study their long‐term safety, including the risk of tumours forming and whether the organoids can be used in different diseases.

### 4.2 BAL Support Systems

BAL systems are used to help patients with acute liver failure when their liver is not working well. They can help patients wait for a liver transplant or help their own liver grow again. Liver organoids are a great source of cells for BAL devices because they are functional and can be expanded.

Yamaguchi et al. created a new device called the UTOpiA system. This device combines two treatments, called granulocyte and monocyte apheresis, with HLA‐depleted hLOs. UTOpiA treatment greatly improved survival in ACLF and ALF rat models, doing better than treatments that only used one drug. Survival improved when coma severity, liver biochemistry, hyperammonaemia and systemic inflammation were reduced [80]. Luo et al. also developed a new type of mini‐liver (RSAL) that can be made quickly using primary hepatocytes. RSALs were able to grow blood vessels within 2 weeks after being transplanted into the mouse mesentery. They successfully protected mice from liver failure caused by a 90%‐hepatectomy, which is a surgery to remove part of the liver. This shows that BAL‐based therapy has potential [36].

### 4.3 Tissue Engineering and Organogenesis

Tissue engineering combines liver organoids (liver cells) with biomaterials (natural or synthetic substances) to create more complex liver tissue constructs. These constructs can be used for transplantation and organ replacement.

Zhu et al. created vascularised liver microtissues by growing liver organoids from stem cells with vascular organoids from hiPSCs in the right proportions. This approach established a complex network of blood vessels similar to the liver's native network, promoting the maturation of liver cells through paracrine signalling from the vasculature [29]. Fan and colleagues developed a new method to create human liver and bile duct cells in large numbers. Special cells were added to human liver models without using extra supplements. Ectopic implants showed that they could fit into the blood vessels and organise themselves into structures that looked like the liver [30]. Branco et al. created a human organoid made up of different types of cells. This organoid shows how the heart, septum and liver form together. By growing these together with cardiomyocyte aggregates, they created a heart organoid with an epicardium‐like layer fully surrounding a myocardium‐like tissue. This shows that organoids can be integrated with other organs [81].

These studies show that combining liver organoids with blood vessels and mesenchymal cells or organoids made from different types of cells can create liver tissue that is structurally and functionally mature. This could be a step forward in transplantation and regenerative medicine.

### 4.4 Gene Correction and Personalised Therapy

Gene editing technologies combined with liver organoids offer new therapeutic strategies for genetic liver diseases. Correcting disease‐causing mutations in patient‐derived organoids followed by autologous transplantation could circumvent immune rejection and provide long‐term therapy [82].

Schene et al. used prime editing for functional repair in patient‐derived disease models, including liver organoids from a patient with Wilson disease (ATP7B). Prime editing was highly efficient and precise, with whole‐genome sequencing revealing no genome‐wide off‐target effects, underscoring its therapeutic potential [37]. Similarly, Rüland et al. CRISPR‐engineered human hepatocyte organoids to model FLC, providing a platform for studying this rare cancer and testing potential treatments [61].

Researchers established a patient‐derived liver cancer organoid biobank, designated LICOB, consisting of miniaturised liver constructs derived directly from individuals with liver cancer [83]. The development of this patient‐derived organoid model system paves the way for advancing personalised therapy for liver cancer and other hepatic disorders—by providing a physiologically relevant ex vivo tool to validate gene editing‐based therapeutic strategies, tailor treatments to individual genetic and phenotypic profiles, and ultimately optimise clinical outcomes for patients.

### 4.5 Clinical Translation and Challenges

Liver organoids show great promise, but there are several hurdles to overcome before they can be used in clinical trials. Safety concerns, such as the risk of tumorigenicity, are very important. This is particularly worrisome for undifferentiated PSC‐derived organoids, which can form teratomas. It is very important to remove all the undifferentiated cells and to test the tumour cells very carefully before transplanting them [84]. Another important thing to consider is how the body might reject the implant. While patient‐specific iPSC‐derived organoids can avoid a person's immune system, the process of creating iPSCs and differentiating organoids takes a long time and a lot of money. This limits how often they can be used in cases of acute liver failure. Organoids created using gene editing to remove immunogenic genes might be a ready solution.

It is also important to consider how well the equipment works and how stable it is over time. Liver organoids usually look more like foetal cells than fully mature adult cells. This might limit how well they work. We still need to learn more about how transplanted organoids can survive and keep working over time. We need to think about the rules and what is right and wrong, especially when it comes to using PSCs and transplanting organoids.

Moreover, liver organoids could provide a new model for understanding how liver dysfunction may exacerbate neurodegenerative diseases, such as Alzheimer's and Parkinson's disease. Since liver‐derived metabolites and toxins can influence brain function, addressing these systemic effects could enhance the treatment of neurodegenerative conditions. Furthermore, the transplantation of functional liver organoids may hold promise for alleviating cognitive and neurological impairments in patients with liver failure and neurodegeneration.

Despite these challenges, liver organoids have a lot of potential in the field of regenerative medicine. As technology improves and new challenges are met, liver organoid transplantation shows promise as an effective strategy for treating end‐stage liver disease. This approach reduces the need for donor organs and improves patient outcomes.

## 5 Challenges and Future Directions

Despite the broad application prospects of liver organoids in basic research and translational medicine, numerous empirical limitations hinder consistent experimental output and clinical transformation. Many cross‐laboratory trials and repeated validation studies have confirmed widespread unreproducible biological phenotypes, including variable hepatocyte maturation degrees, random epigenetic drift during long‐term passaging and unstable paracrine signalling in multi‐cell co‐culture systems. Unresolved technical bottlenecks further exacerbate experimental inconsistency, such as batch variation of animal‐derived extracellular matrix, low controllability of vascular network construction, and the absence of unified quality control standards. Many representative attempts, including high‐throughput drug screening for metabolic liver diseases and viral infection modelling, have shown poor repeatability across parallel batches and independent laboratories [39, 85]. This subsection complements the one‐sided optimistic descriptions of organoid advantages in previous chapters, systematically expounds the practical defects of current liver organoid platforms and improves the objectivity and academic rigour of this review.

Liver organoid technology has a lot of potential in disease modelling and regenerative medicine, but there are still several big challenges. It is very important to deal with these issues if we want to move this technology closer to being used in medical treatments and other applications. This section talks about the main problems with current liver organoid technology and suggests possible future improvements (Figure 4).

**FIGURE 4:**
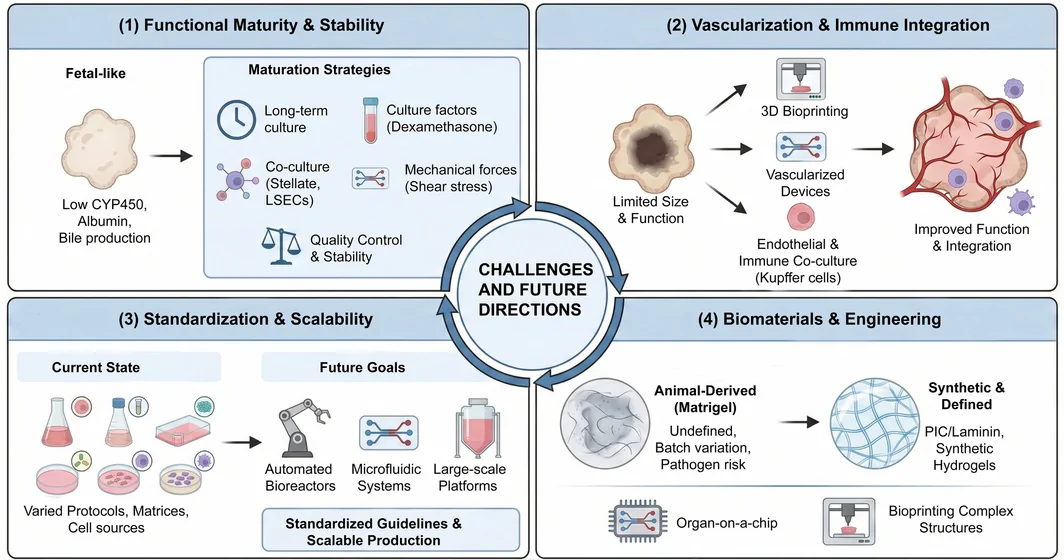
Challenges and future directions in liver organoid technology. (1) Functional maturity and stability: Native liver organoids display foetal‐like low hepatic synthetic function; maturation approaches including long‐term culture, stromal co‐culture, chemical induction and mechanical stimulation improve differentiation and functional stability. (2) Vascularisation and immune integration: Poor vascularisation limits organoid size and function; 3D bioprinting, vascularised culture devices and endothelial–Kupffer co‐culture build vascular‐immune networks to boost tissue integration. (3) Biomaterials and engineering: Traditional animal‐derived matrices have batch variation and biosafety risks; defined synthetic hydrogels, organ‐on‐a‐chip and bioprinting are developed for biomimetic complex hepatic construction. (4) Standardisation and scalability: Current culture protocols lack uniformity in matrices and cell sources; automated bioreactors, microfluidics and unified guidelines realise standardised large‐scale organoid production.

### 5.1 Functional Maturity and Long‐Term Stability

One problem with liver organoids right now is that they do not work as well as they should. Most models retain foetal‐like transcriptional signatures rather than phenotypes of fully mature adult liver cells. This functional immaturity is reflected by multiple quantitative defects: the activity of core drug‐metabolising CYP450 enzymes only reaches 40%–105% of primary human hepatocytes, among which CYP2D6 and CYP1A2 show the lowest activity at merely 40%–60% [75, 86], albumin secretion and urea synthesis drop to 60%–80% and 50%–70% of primary hepatocyte levels, respectively [18, 87], bile canaliculus formation and ammonia clearance capacity are also markedly compromised.

Research by Hendriks et al. showed that liver cells from foetuses and adults are different when they grow into organoids. In PHH organoids, adult genes initially mimic foetal genes but quickly become imbalanced, with too much cell growth and fat production, which causes liver damage and stops growth. They found that adding IL6 and activating the metabolic regulator FXR together prevented liver fat buildup and promoted liver cell growth [39]. Here are some strategies to help liver organoids develop their abilities: (1) increasing the culture time to allow for more complete differentiation; (2) changing the culture conditions to include factors that promote maturation (like dexamethasone, insulin and thyroid hormone); (3) growing together with other cells, such as stellate cells and LSECs; (4) using mechanical forces, like fluid shear stress, to mimic blood flow; and (5) designing a 3D structure that encourages cell polarity and bile canaliculi network formation.

Another challenge is making sure it is stable over time. Organoids may change in their genes or epigenetics (chemical changes that affect how genes are expressed) when they are cultured for a long time. This can affect how they work and how reliable they are. It is very important to have quality control standards and to regularly check that the genome is stable. This helps to make sure that experiments can be repeated and that they are safe for patients.

### 5.2 Vascularisation and Immune Integration

Not enough blood vessels can limit the size and function of liver organoids. In the liver, hepatocytes have many blood vessels, and each cell is in contact with at least two sinusoids. This allows for efficient exchange. In a lab setting, the absence of a vascular network causes cell death in the centre of the sample, limiting its size to 200–500 μm and impeding waste removal. Researchers are exploring different ways to grow blood vessels. First, organoids are grown with endothelial cells to help them form a natural blood vessel network. Second, special devices are used to create a culture environment that resembles blood flow. Third, organoids are printed in 3D using a bioprinter, which connects them to channels made by vascular networks. Finally, the organoids are given time to integrate with the blood vessels of the host's body after a transplant.

The researchers found a group of factors that can cause blood vessels to form in an organised way in the heart tissue samples. They also found that this method can be used to make blood‐filled liver tissue samples. These findings suggest that blood vessel formation in different organs may follow a similar, conserved developmental programme [88]. Zhu et al. successfully created vascularised liver microtissues by co‐culturing organoids, showing improved functional maturation [29].

It is very important to consider immune integration when transplanting organoids. The liver has different immune cells, like Kupffer cells. These cells help to protect the body and accept different things. By adding immune cells to liver organoids, we can better understand the immune system and diseases like hepatitis and fibrosis [89].

### 5.3 Standardisation and Scalability

The lack of standardisation is a significant challenge. The way organoids are made today varies a lot from lab to lab. This is because they use different cell sources, media formulations and matrices. This leads to different results and problems when trying to reproduce experiments. It is very important to create a set of rules for creating, understanding and growing organoids.

Marsee et al. came up with a definition and name for these cells and tissues, and they created guidelines for creating, understanding and improving these systems in the future. They created a simple classification system and naming scheme that experts agreed on [85].

Another challenge for using these in a clinical setting is that they cannot be produced in large enough quantities. Making enough organoids for transplantation or high‐throughput drug screening requires ways to produce them that are easy to scale up and not too expensive [90]. Making organoids is hard work, and it is hard to make more than a few at a time. Researchers are developing microfluidic systems, bioreactors and large‐scale culture platforms to address this. Abbasalizadeh et al. created a microfluidic system that can be scaled up to produce cell‐loaded microcapsules with a biodegradable hydrogel shell. This shell supports cell growth, self‐condensation and the creation of liver organoids through self‐organisation [91].

### 5.4 Biomaterials and Engineering Strategies

The use of animal‐derived materials (like Matrigel) limits the use of organoid technology in clinical settings. These materials are not well defined, can vary from batch to batch and may carry germs. Researchers are working to create special materials to support organoids as they develop.

Researchers have created different types of synthetic hydrogels for culturing liver organoids. A new type of gel was described. It is made of PIC and laminin‐111. It can be used to grow HLOs. PIC is a man‐made polymer that forms a thermosensitive hydrogel, which makes it easy to handle and attractive for clinical use [19]. Sorrentino et al. used special gels to make liver cells from mice and humans without using any animal products [27].

Technologies like organ‐on‐a‐chip and bioprinting are being used to make organoids more complex and functional. Tao et al. created a small‐scale model of multiple organs to study the human liver‐islet axis in normal and diseased states [33]. Urciuolo et al. developed a special technique that uses a hydrogel‐in‐hydrogel live bioprinting approach to create instructive hydrogel elements within organoid cultures that already exist [92].

### 5.5 Ethical and Regulatory Considerations

There are ethical concerns about organoid technology, especially when it comes to brain organoids and human‐animal chimeras. Liver organoids cause fewer ethical concerns, but we still need to think about the use of hPSCs, especially when we're dealing with germline or embryonic materials. While it is unlikely that liver organoids will develop consciousness or sentience, this possibility should be considered as organoids become more complex [93].

As organoids are increasingly used in modelling diseases like neurodegenerative disorders, ethical considerations will also extend to how they might impact brain function and whether such models could unintentionally cross ethical boundaries in terms of consciousness.

Current single‐type organoid models are gradually being replaced by multi‐organ co‐culture systems to recapitulate inter‐organ crosstalk under physiological and pathological conditions; among these hybrid platforms, co‐cultures integrating liver organoids and brain organoids have emerged as powerful tools to dissect liver‐brain axis dysfunction underlying neurodegenerative and metabolic encephalopathic diseases. This combinatorial model introduces unique consciousness‐associated ethical risks absent from isolated liver or brain organoid culture alone. The liver organoid provides metabolic support, toxin clearance and circulating cytokine signalling to sustain prolonged maturation of neural circuits within brain organoids, facilitating the formation of more sophisticated, interconnected neuronal networks with synchronised electrophysiological activity analogous to immature foetal neural tissue [94]. Such enhanced neural complexity elevates the theoretical risk of generating minimal phenomenal sentience or subjective experiential capacity within the integrated assembloid system, a hazard amplified by the prolonged survival window enabled by hepatic metabolic co‐support [95]. Traditional ethical risk assessments separate hepatic and neural organoid systems, failing to anticipate synergistic maturation effects from liver‐brain co‐culture; bioethicists have therefore advocated a precautionary neuroethical framework that mandates tiered risk classification for all multi‐organ assembloids containing functional brain components, with dedicated oversight committees to monitor neural circuit complexity and electrophysiological markers suggestive of primitive awareness before and during all co‐culture experiments [96]. Without targeted ethical governance for liver‐brain hybrid organoids, researchers may inadvertently construct integrated biological systems that breach widely accepted moral thresholds regarding the creation of potentially sentient in vitro neural tissue, creating unregulated grey zones between basic disease modelling and ethically contentious organoid intelligence research.

Furthermore, the potential for liver organoid transplantation to treat both liver failure and neurodegenerative diseases raises additional concerns about the long‐term effects on patients, including whether these transplants could alter brain function or exacerbate existing neurological issues.

Regulations need to be updated to reflect the unique features of organoid technology. Organoids can be regulated as cell therapy products or medical devices, depending on how they are used. Before organoid transplants can be used, we need to make sure they are safe and effective. This will require a lot of research and testing [97].

Schneemann et al. discussed the ethical challenges of paediatric liver organoid transplantation, questioning the acceptability of including children with liver disease in the first‐in‐human trial of this new intervention. They emphasised the importance of carefully weighing potential benefits against risks when advancing organoid‐based therapies [98].

## 6 Conclusion and Perspective

Liver organoid technology has emerged as a pivotal tool in liver research, with remarkable potential in disease modelling and regenerative medicine. By recreating the complex structure and function of the human liver in a lab, it performs better than traditional 2D cell cultures and animal models. This new method offers a unique way to study liver development, disease mechanisms and how drugs affect the liver.

It has been effective in modelling various liver diseases and enabling personalised medicine research. It also shows promise as a cell source for transplantation and BAL systems [99]. However, the technology faces challenges, including its limited functionality, insufficient blood vessel growth, lack of standardisation and difficulties in producing it on a large scale. To address these challenges, we need to work together across different fields of expertise.

We expect future advancements in four key areas: making things more complex by integrating multiple cells and using mechanical stimuli, creating standard ways to do things so they can be reproduced safely, developing platforms for large‐scale production and advancing clinical translation. As these challenges are resolved, liver organoids will transform basic liver research and clinical practice, leading to progress in precision and regenerative medicine to improve outcomes for liver disease patients.

## Author Contributions

Shuliang Niu, Ting Zhang and Tiepeng Wang conceptualised, supervised and revised the manuscript. Tiepeng Wang and Xiaotian Qu conducted the literature review and drafted the paper. Jinhong Si and Junkang Meng assisted with data analysis, manuscript preparation and manuscript revision. Shuliang Niu and Ting Zhang provided funding and project administration. All co‐authors have reviewed and approved this version of the manuscript.

## Funding

This work was supported by the National Natural Science Foundation of China (No. 81460202), the Fundamental Research Funds for Universities in Xinjiang (No. XJEDU2026Z018), the Karamay Key R&D Program Projects (No. 2025BA0090), the High‐Level Talent Research Startup Program of Xinjiang Second Medical College (No. XGGK2025‐02), the Xinjiang Second Med Coll Res Innov Team Program (No. KT202603) and the Undergraduate Innovation and Entrepreneurship Training Program of Xinjiang Second Medical College (No. 202513560001).

## Conflicts of Interest

The authors declare no conflicts of interest.

## Data Availability

Data sharing is not applicable to this article as no datasets were generated or analysed during the current study.
